# The Health Benefits of Dietary Fibre

**DOI:** 10.3390/nu12103209

**Published:** 2020-10-21

**Authors:** Thomas M. Barber, Stefan Kabisch, Andreas F. H. Pfeiffer, Martin O. Weickert

**Affiliations:** 1 Warwickshire Institute for the Study of Diabetes, Endocrinology and Metabolism, University Hospitals Coventry and Warwickshire, Clifford Bridge Road, Coventry CV2 2DX, UK; 2 Division of Biomedical Sciences, Warwick Medical School, University of Warwick, Coventry CV4 7AL, UK; 3 NIHR CRF Human Metabolism Research Unit, University Hospitals Coventry and Warwickshire, Clifford Bridge Road, Coventry CV2 2DX, UK; 4 Department of Clinical Nutrition, German Institute of Human Nutrition Potsdam-Rehbrücke, Arthur-Scheunert-Allee 114-116, 14558 Nuthetal, Germany; Stefan.Kabisch@dife.de (S.K.); afhp@dife.de (A.F.H.P.); 5 Deutsches Zentrum für Diabetesforschung e.V., Geschäftsstelle am Helmholtz-Zentrum München, Ingolstädter Landstraße, 85764 Neuherberg, Germany; 6 Department of Endocrinology, Diabetes and Nutrition, Campus Benjamin Franklin, Charité University Medicine, Hindenburgdamm 30, 12203 Berlin, Germany; 7 Centre for Sport, Exercise and Life Sciences, Faculty of Health & Life Sciences, Coventry University, Coventry CV4 7AL, UK

**Keywords:** dietary fibre, gut microflora, obesity

## Abstract

Background: Dietary fibre consists of non-digestible forms of carbohydrate, usually as polysaccharides that originate from plant-based foods. Over recent decades, our diet within Westernised societies has changed radically from that of our hominid ancestors, with implications for our co-evolved gut microbiota. This includes increased ingestion of ultra-processed foods that are typically impoverished of dietary fibre, and associated reduction in the intake of fibre-replete plant-based foods. Over recent decades, there has been a transformation in our understanding of the health benefits of dietary fibre. Objective: To explore the current medical literature on the health benefits of dietary fibre, with a focus on overall metabolic health. Data Sources: We performed a narrative review, based on relevant articles written in English from a PubMed search, using the terms ‘dietary fibre and metabolic health’. Results: In the Western world, our diets are impoverished of fibre. Dietary fibre intake associates with overall metabolic health (through key pathways that include insulin sensitivity) and a variety of other pathologies that include cardiovascular disease, colonic health, gut motility and risk for colorectal carcinoma. Dietary fibre intake also correlates with mortality. The gut microflora functions as an important mediator of the beneficial effects of dietary fibre, including the regulation of appetite, metabolic processes and chronic inflammatory pathways. Conclusions: Multiple factors contribute to our fibre-impoverished modern diet. Given the plethora of scientific evidence that corroborate the multiple and varied health benefits of dietary fibre, and the risks associated with a diet that lacks fibre, the optimization of fibre within our diets represents an important public health strategy to improve both metabolic and overall health. If implemented successfully, this strategy would likely result in substantial future health benefits for the population.

## 1. Introduction

In our modern-day, 21st century world, chronic diseases predominate. Underlying much of this chronic disease burden are pathological pathways that implicate inflammation and metabolic dysfunction (including insulin resistance). In recent decades, much evidence supports an important role for our lifestyles in the development of such inflammatory and dysmetabolic processes, such as, for example, our sleep, physical activity and diet. Such lifestyle factors also contribute towards weight gain and obesity, which represent a particularly important contributor to chronic ill health, including >50 medical conditions (such as type 2 diabetes mellitus (T2D), dyslipidaemia, hypertension, obstructive sleep apnoea and cardiovascular disease). Global obesity prevalence has tripled over the last half century, with current rates of obesity and overweight affecting 650 million and 1.9 billion people respectively [1,2]. In addition to multi-morbidity, obesity also associates with premature mortality as evidenced by data from the Framingham Heart Study [3]. Obesity has a substantial and diverse impact on psycho-social functioning [4], work productivity [5] and global healthcare expenditure [6].

Important advances have been made in recent years regarding our understanding of appetite and metabolic regulation [7], metabolic surgical [8] and medical therapeutic approaches to obesity [9]. However, regardless of therapeutic choice for obesity management, ultimately, weight loss stems from behavioural change at an individual level [10]. At the heart of such behavioural change lies dietary modification. Despite this insight, however, it is remarkable how little we know about our diet. Nutritional studies are notoriously difficult to execute and interpret for a variety of reasons that include self-reporting of food intake [11], multiple confounding factors (such as variations in genetic, metabolic and gut microbial factors), impaired compliance with dietary changes and the difficulties associated with the study of macronutrient changes in isolation (with inevitable consequences for other ingested macronutrients). All of these factors conspire to create much uncertainty regarding the optimal dietary needs for the individual. To compound this uncertainty, the popular media is littered with an effusion of fad diets with phony promises of long-lasting weight loss and health, often based on little if any proper scientific evidence and rigour. It is little wonder that patients and many healthcare professionals are confused about the optimal diet to follow and advocate. In response, esteemed societies and governments around the world have tended to offer generic dietary advice on a population-based level. For decades, much focus has rested with advice to adopt a ‘low-fat’ diet [12] that, more recently, has shifted towards a ‘low-carbohydrate’ diet [13] (with associated examples of ‘sugar-tax’ and limitations on food advertising for children).

In this narrative review, we focus on dietary fibre, a macronutrient that has perhaps not received as much attention as its more attractive counterparts, fat and carbohydrates. Our objective was to explore the current medical literature on the health benefits of dietary fibre, with a focus on overall metabolic health but also on gut motility, gut microbiota, chronic inflammation, mental health, cardiovascular disease, colorectal carcinoma and mortality. We also provide a suggested strategy for how we can optimise dietary fibre intake within the population in the context of our fibre-impoverished modern-day world.

## 2. Methodology

We performed a narrative review of the current literature. We used PubMed for this purpose. The search terms were as follows: ‘dietary fibre and metabolic health’. We only considered articles written in English, with no restrictions on the date of publication.

## 3. Dietary Fibre

The chemical classification of carbohydrates stems from molecular size. Whilst sugars (1–2 monomers) and most oligosaccharides (3–9 monomers) are digestible, polysaccharides (≥10 monomers) are usually non-digestible [14]. Although technically a type of carbohydrate, it is their non-digestibility (in turn relating to their molecular size) that provides the quiddity of dietary fibre. Accordingly, the European Food Safety Authority (EFSA) defines dietary fibre as non-digestible carbohydrate plus lignin. EFSA provide a long list of substances that constitute dietary fibre, including non-starch polysaccharides, cellulose, pectins, hydrocolloids, fructo-oligosaccharides and ‘resistant starch’ [14].

The classification of dietary fibre also stems from water solubility. Broadly, there are two main types of dietary fibre: soluble and insoluble. The main sources of soluble fibre are fruits and vegetables. Conversely, cereals and whole-grain products provide sources of insoluble fibre [15]. However, most naturally available high-fibre foods contain variable amounts of both soluble and insoluble fibre [15]. Although fermentation (through the action of the gut microbiota) of most dietary fibre occurs within the gastrointestinal tract to some extent, the fermentation of soluble fibre tends to occur more readily than that for insoluble cereal fibres [15].

Hippocrates first described the laxative effects of coarse wheat [14]. Interest in dietary fibre was piqued in the 1920s through publications from Kellogg, including the health benefits of bran (such as laxation, stool weight and prevention of disease) [14,16]. During much of the middle part of the 20th century, dietary fibre experienced a lull period that re-awakened in the 1970s through publications from Burkitt, to suggest protective effects of dietary fibre from Diabetes Mellitus, colonic cancer and obesity [14,17]. Since then, research on the health benefits of dietary fibre has continued apace, with transformation of our understanding of such benefits over that time [18].

## 4. Dietary Fibre Intake in the Western World

Current recommendations for dietary fibre intake for adults in most European countries and in the US are between 30–35 g per day for men and between 25–32 g per day for women [19]. Based on such recommendations, it is important to ascertain the actual dietary fibre intake for adults in these countries. Stephen and colleagues recently reported a comprehensive review of dietary fibre intake in European countries, including data from nearly 140,000 individuals covering a broad age range from early childhood to older adulthood [19]. Overall, dietary fibre intake for adults living in European countries was 18–24 g per day for men and 16–20 g per day for women, with grain products (including bread) providing the largest source of dietary fibre [19]. There was little variation in dietary fibre intake between European countries, and data were broadly similar between adult age groups [19]. Comparison with data from the National Health and Nutrition Examination Survey (NHANES) showed that on average, dietary fibre intake in European countries was higher than in North America. Based on these data, it appears that within Europe and the US, dietary fibre intake is around one third below the recommended level. Stated differently, within the Western world, most of us should increase our dietary fibre intake by around 50% compared to our current intake.

Following this overview of the nature of dietary fibre, the recommended intake for adults and the relative impoverishment of fibre within our modern-day Westernised and highly processed diets, it is important to explore the potential beneficial health effects of dietary fibre, and the supporting evidence.

## 5. The Benefits of Dietary Fibre

As alluded to earlier, there was a re-awakening of scientific interest in dietary fibre in the 1970s, following published studies by Burkitt [19,20]. Since then, much scientific evidence has accumulated on the association of dietary fibre with body weight and overall metabolic function (including effects on glucose and lipid regulation and insulin sensitivity). Perhaps at least in part stemming from the known associations of dietary fibre intake with a healthy gut microbiome, favourable body weight and overall metabolic health, dietary fibre intake also associates with a reduced risk for the development of cardiovascular disease and mortality. There have also been descriptions of further health benefits of dietary fibre, including reduced risk of malignancy and improved colonic health. In this section, we provide a summary of the main health benefits of dietary fibre and the risks associated with a lack of dietary fibre, with a focus on abdominal adiposity and overall metabolic health [15].

### 5.1. Gut Motility

Of all the beneficial effects of dietary fibre, perhaps the most widely known and appreciated is the effect on gut motility and prevention of constipation. Many studies support such an effect, which appears incontrovertible based on the available evidence. In one randomised controlled double-blind trial on the effects of ‘vege-powder’ (consisting of chicory, broccoli and whole grains) on constipation alleviation in >90 participants, compared with the control group, those who received vege-powder had significant improvements in symptoms of constipation (including stool hardness, defecation frequency and straining to defecate) at 2 and 4 weeks [21]. Further evidence to support the clinical utility of dietary fibre as an effective treatment of constipation stems from a systematic review reported by Rao et al. [22]. Evidence was sought for dietary fibre intake and restrictions in fermentable oligosaccharide, disaccharide, monosaccharide and polyol (FODMAP-restricted diet) in the management of chronic constipation and irritable bowel syndrome (IBS) [22]. For chronic constipation, dietary fibre was beneficial in five of the seven studies examined, and all three of the studies of IBS-associated constipation. The FODMAP-restricted diet also appeared to improve overall IBS symptoms [22]. Current evidence would appear to support the beneficial effects of dietary fibre on gut motility and as an effective management strategy for both the prevention and treatment of constipation.

### 5.2. Body Weight and Abdominal Adiposity

Dietary fibre supplementation may facilitate weight loss through reduced frequency of eating and food consumption [23]. To test this hypothesis, Solah and colleagues performed a three-arm, parallel, blind, randomised controlled trial with either 4.5 g of PolyGlycopleX (PGX) as softgel, 5 g PGX as granules or 5 g of rice flour (RF) as a control [23]. Compared with the RF control, in the PGX granules group, there was a significant reduction in waist circumference (−2.5 cm), body weight (−1.4 kg), Body Mass Index (BMI) (−0.5 kg m^−2^) and number of eating occasions and intake of grain food [23]. However, in other studies, the effects of fibre consumption on weight loss and changes in anthropometric markers were, at best, minor, or no significant and clinically relevant effects were observed [24,25].

To explore further the association of dietary fibre intake (in the form of pulse consumption) on body weight and composition, Kim and colleagues reported on a systematic review and meta-analysis of 21 randomised controlled trials including 940 participants [26]. Pooled data showed an overall significant reduction in body weight of −0.34 kg for diets containing dietary pulses versus diets without pulse intervention (median duration of 6 weeks). Data from six of the trials also suggested an association of dietary pulse consumption with reduced body fat percentage [26]. In a separate systematic review and meta-analysis of the literature on the effects of viscous fibre ingestion on body weight and composition from 62 trials and 3877 participants, there was a similar significant association with weight loss of −0.33 kg [27]. Finally, in the ‘Preventing Overweight Using Novel Dietary Strategies’ (POUNDS Lost) study, in 345 participants who consumed a calorie-restricted diet (−750 kcal per day) for 6 months, the most successful predictor of reduction in body weight from a variety of anthropometric and dietary factors was intake of dietary fibre [28]. Furthermore, dietary fibre was also strongly associated with adherence to the macronutrient prescriptions within the calorie-restricted diets [28].

Based on current published evidence, dietary fibre appears to associate only with small improvements in body weight, and evidence for changes in body composition (including fat mass) is less clear. A small reduction in abdominal adiposity (as reflected by changes in waist circumference) likely also associates with dietary fibre intake and is probably reflective of changes in overall body weight. Although some evidence suggests that reduction of food intake (both frequency and size of meals) associates with increased fibre intake, future studies should explore the mechanisms that mediate effects of dietary fibre on body weight and composition (for example, through effects on appetite regulation). Finally, the studies reported to date on the association between dietary fibre and body weight and composition are relatively short-term or animal-based, with very few long-term studies available [29]. Future studies should also focus on the longer-term benefits of dietary fibre on weight loss and maintenance of body weight. The association of dietary fibre intake with only small improvements in body weight suggests that dietary fibre *per se*, is not a realistic solution for effective weight reduction. Intake of dietary fibre for body weight *maintenance* would be of interest, although there are few published data on this aspect of weight management. However, regardless of changes in body weight, intake of a high-fibre diet may be particularly relevant for the obese population due to the increased metabolic and cardiovascular risk associated with obesity and the clear metabolic benefits of dietary fibre, outlined below.

### 5.3. Insulin Sensitivity and Metabolic Health

There is much evidence in the literature to support an association between dietary fibre intake and insulin sensitivity [30,31,32,33,34,35]. Our own group published relevant evidence from an interventional trial using data from ProFiMet, the most highly-phenotyped cohort [36]. In this study, 111 overweight adults with features of the metabolic syndrome were randomly assigned to one of four 18-week isoenergetic diets, including control, high cereal fibre (HCF), high protein (HP) and mixed high cereal fibre and protein (Mix) groups. Amongst the 84 participants who completed the dietary intervention, it was demonstrated that compared with the HP diet group, insulin sensitivity was significantly (25%) higher in the HCF group [36]. Furthermore, HCF intake prevented the reduction of insulin sensitivity upon increased protein intake [36]. The attenuated effects of HCF on insulin sensitivity at 18 weeks likely related to diminished adherence to the HP diet [36].

In addition to data from interventional trials, there have also been reports from observational studies to corroborate the metabolic benefits of dietary fibre. In one such study, Morimoto and colleagues reported on a study on a Japanese cohort (*n* = 190) without T2D to explore the metabolic effects of dietary fibre intake in the context of a diet and exercise program [37]. Increases in the ratio of dietary fibre to carbohydrate intake during the 5-month period of the study associated significantly with a reduction in HbA1C [37]. The authors suggested the potential utility of an increased dietary ratio of fibre to carbohydrate in the prevention of T2D [37]. In a separate study from Mexico on 217 adolescents, it was demonstrated that those with the highest dietary fibre intake had lower odds of homeostasis model assessment of insulin resistance (HOMA-IR) >2.97 (OR 0.34; 95% CI 0.13–0.93), following adjustments for age, sex, body fat percentage and intake of saturated fatty acids [38]. Finally, in a recent systematic review and meta-analysis on the effects of dietary fibre and whole grains in the management of Diabetes Mellitus, there was an association of high-fibre diet with improved insulin sensitivity, including many other aspects of metabolic health, such as HbA1C, lipid profile, body weight and C-reactive protein [39]. There is, however, a deficiency of published longer-term controlled studies (>12 months) on the metabolic effects of increasing dietary fibre intake [39] (with a notable exception [35]), and this should be a focus for future research in this field.

A high intake of soluble dietary fibre appears to have additional metabolic benefits, including improved glycaemic index of carbohydrate-rich foods and lipid profiles [33,40,41]. However, in this context, it is remarkable and surprising that it is mainly the consumption of insoluble cereal dietary fibre and whole grains (and not soluble fibre) that associate consistently with a reduced risk for the development of T2D in large prospective cohort studies [15,42,43]. Other effects of dietary fibre that may also have an impact on overall metabolic health include the release of various gut hormones [44,45,46,47,48,49], adipokines [50], bile acids [51] and the metabolic signatures of amino acids [52]. Based on the current scientific evidence from prospectively designed and controlled studies, dietary fibre does appear to associate with improvements in insulin sensitivity and overall metabolic status. Future studies should focus on the underlying mechanisms implicated, which mediate the metabolic benefits of dietary fibre.

### 5.4. Gut Microflora and Metabolites

The gut microflora consists of around 100 trillion microbes that co-evolved with our hominid ancestors over millions of years [53]. In recent decades, there has been a transformation of our understanding of the gut microflora. A healthy and diverse gut microflora underlies normal physiology, including normal immune development, metabolic and appetitive pathways and even regulation of normal mental and emotional functioning [53]. Gut dysbiosis underlies much of 21st century chronic ill health through effects on chronic inflammatory pathways and immune dysfunction, the latter resulting in atopy, food intolerances and autoimmune conditions. Fortunately, our gut microflora is modifiable through lifestyle factors, primarily our diet [53]. We can all, therefore, improve our future health prospects through improving our gut flora. One excellent way to achieve this is through optimizing our dietary fibre intake. Much of our evidence for the role of dietary fibre on the gut microbiota and the implications for health stems from rodent-based studies. These include the effects of dietary fibre intake on colonic health. In one such study using a gnotobiotic mouse model, in which there was colonization with harvested human gut microbiota, chronic dietary fibre deficiency resulted in the gut microbiota using host-secreted mucus glycoproteins as an alternate nutrient source [54]. There was subsequent erosion of the colonic mucus barrier with greater epithelial access and predisposition to lethal colitis [54]. It is likely that in humans, dietary fibre also plays a protective role for the intestinal barrier and overall colonic health.

Through direct interaction with our gut microbes, dietary fibre also influences microbial ecology and enhances the production of key microbial metabolites, such as short-chain fatty acids (SCFAs) that, in turn, promote our overall health and wellbeing [55]. Anaerobic microbes within the caecum produce SCFAs during fermentation of dietary fibre. In addition to providing a source of energy for colonocytes, SCFAs also pass through the colonic epithelium into the bloodstream and influence lipid, glucose and cholesterol metabolism through effects on G protein-coupled receptors [56]. Rodent-based studies also suggest that SCFAs may influence gut motility [57], suppress appetite (through enhanced incretin [glucagon-like peptide-1, GLP-1] release) and enhance insulin sensitivity [58]. Human-based studies corroborate the beneficial effects of SCFAs, including the effects of propionate (a common SCFA produced by human gut microbiota) on enhanced incretin response (including plasma Peptide YY [PYY] and GLP-1 excursions), weight loss, reduced intra-abdominal adipose tissue volume and intra-hepatocellular lipid content and preservation of insulin sensitivity [59]. The interactions within the gut microbiota–brain axis are likely to be complex and multi-directional [60,61] and implicate the release of by-products from gut microbes, including SCFAs, secondary bile acids and tryptophan metabolites [60,62]. Such molecules may promote signalling via enterochromaffin cells, enteroendocrine cells and the mucosal immune system. SCFAs may also cross the blood–brain barrier and exert direct effects on hypothalamic regulation of metabolic pathways and appetite [63,64]. However, it remains unclear whether dietary fibre-induced changes in SCFAs are indeed a key factor conveying the beneficial metabolic effects of a high fibre intake [65]. In this context, it is interesting that insoluble cereal fibres from wheat or oat extracts are non-fermentable in vivo and in vitro [65], whereas it is this type of dietary fibre (including whole grain products), and not the soluble and highly fermentable fibre types, that mainly appears to improve insulin resistance and reduce the risk of developing T2D [25]. Perhaps one explanation for the metabolic benefits of insoluble cereal fibres (including alteration of metabolite profiles [52,65]) stems from their association with increased faecal bulk and, therefore, microbial mass.

### 5.5. Chronic Inflammation

It has been postulated that low intake of dietary fibre is a risk factor for both local and systemic chronic inflammation [66,67]. The current dogma suggests that limited dietary fibre intake stymies the establishment and maintenance of a healthy, viable and diverse colonic microbiota that, in turn, limits the local production of SCFAs, including butyrate. Signalling pathways that implicate nuclear factor kappa-B (NF-ĸB) and inhibition of deacetylase influence inflammatory processes both locally (including gut-wall leakiness and colonic inflammation in patients with inflammatory bowel disease [68]) and systemically, and both are likely influenced by levels of butyrate within the colon [66]. Furthermore, butyrate may improve oxidative stress within the colon through effects on gene expression implicated in glutathione and uric acid metabolism [69].

In support of a role for dietary fibre in influencing inflammatory pathways, Miller and colleagues reported a cross-sectional study on >140 overweight Hispanic and African-American adolescents [70]. This study revealed that the tertile with the highest consumption of dietary fibre intake (compared with the tertile with the lowest dietary fibre intake) had significantly lower plasma markers of inflammatory status, including 36% and 43% lower levels of plasminogen activator inhibitor-1 (PAI-1) and resistin, respectively, with similar data for insoluble fibre [70]. In a much larger study from the UK, Gibson and colleagues reported on data from the Airwave Health Monitoring Study, a cross-sectional analysis on 6898 participants with 7-day food records [71]. Data from this study revealed a significant inverse linear trend across fifths of total fibre intake and consumption of fibre from fruit with C-reactive protein (CRP, a plasma measure of general inflammatory status) and BMI, percentage body fat and waist circumference [71]. In this study, given the association of dietary fibre intake with body fat percentage, it is possible that the favourable effects of dietary fibre on inflammatory status (indicated by plasma level of CRP) are actually mediated, at least in part, by changes in body composition rather than a direct effect of dietary fibre. In a further study, Kabisch and colleagues demonstrated an interventional interaction effect of dietary fibre supplementation on inflammation [35]. Interestingly, whilst the effect size for the anti-hyperglycaemic properties of insoluble dietary fibre seemed to depend mainly on the prevailing metabolic state, the anti-inflammatory effect of the particular supplement used in this study related primarily to the presence or absence of obesity [35].

Given the likely effects of dietary fibre on colonic microbiota diversity and production of SCFAs outlined above, and the known effects of butyrate in the mediation of inflammatory pathways, it is entirely plausible, and indeed likely, that dietary fibre does have at least some influence on inflammatory status both within the colon and systemically. The mechanisms implicated should form a focus for future research. Given the multiple possible beneficial effects of dietary fibre explored in this section and the complexity of the implicated mechanisms (including involvement of the colonic microflora), identifying the actual mechanisms that mediate the anti-inflammatory effects of dietary fibre will likely be challenging and necessitate a variety of approaches, including prospectively designed Randomised Controlled Trials (RCTs) and rodent-based mechanistic studies.

### 5.6. Depression

Dietary fibre intake appears to associate with risk for the development of depression. Although the underlying mechanisms remain incompletely understood, it has been hypothesised that inflammation may mediate the link between dietary fibre and depression, and that the association between a high-fibre diet and a reduction in inflammatory compounds may alter the concentrations of certain neurotransmitters that, in turn, could reduce the risk for the development of depression [72]. Consistent with a role for the gut microbiota in the mediation of fibre-effects on mental health, a meta-analysis of controlled clinical trials showed a small but significant effect of probiotics on depression and anxiety [73]. Furthermore, proof of the concept that a healthy diet improves depressive symptoms was provided in the SMILES trial, in which a modified Mediterranean diet (including nutrition counselling sessions) in adult patients with poor quality diets and major depressive disorders was shown to associate with improvements in depressive symptoms compared with the control group [74]. Given the association of poor diet and obesity with depression and other mental health problems, and the data outlined here, it is important for future studies to provide insights into the mechanisms linking our diet (including dietary fibre) with our mental health. Future guidance on the prevention and management of depression and other mental health disorders may also include a high-fibre diet as an important factor to consider.

### 5.7. Cardiovascular Disease

Given the association between dietary fibre with favourable insulin sensitivity, body composition, appetite regulation and diversity and viability of the gut microflora, it is important to address whether these associations also translate into an effect on overall rates of cardiovascular disease (CVD). Threapleton and colleagues published a systematic review and meta-analysis of the available literature on this topic [75], with inclusion of 22 prospective cohort studies reporting on associations between dietary fibre intake and coronary heart disease or CVD, with a minimum follow-up period of 3 years. There was an inverse association between total dietary fibre intake (including insoluble fibre and fibre from cereal and vegetable sources) and risk of CVD (risk ratio 0.91 per 7 g/day (95% CI 0.88–0.94)), and coronary heart disease (risk ratio 0.91 per 7 g/day (CI 0.87–0.94)) [75]. Fruit fibre was only associated inversely with risk for CVD [75].

Consistent with a beneficial effect of dietary fibre on overall risk for CVD, data from a more recent study have shown an association between consumption of ultra-processed foods (with typical impoverishment of dietary fibre) and increased CVD risk. Srour and colleagues reported on a large prospective population-based study from France (the ‘NutriNet-Santé cohort’), with >105,000 adult participants reporting repeated 24-h dietary records [76]. Over a median follow up of 5.2 years, intake of ultra-processed food was associated with a higher risk of overall CVD, coronary heart disease and cerebrovascular disease. Interestingly, these associations remained statistically significant following adjustment for intake of other macronutrients, including dietary fibre [76].

Finally, Kim et al. reported a meta-analysis from 15 prospective cohort studies on the association between dietary fibre intake and mortality from CVD and all cancers [77]. Pooled risk ratio for CVD mortality (based on the highest versus the lowest categories of dietary fibre intake) was 0.77 (95% CI: 0.71–0.84) [77]. There were also associations between increased dietary fibre intake and lower risk of mortality from coronary heart disease and all cancers. A dose–response meta-analysis also revealed a pooled relative risk reduction by 9% for CVD mortality for an increment of 10g/day of dietary fibre intake [77].

Based on the available published literature, including meta-analyses and large population-based studies, there does appear to be an association between dietary fibre intake and both risk and mortality from CVD (including coronary heart disease and cerebrovascular disease). Evidence also suggests an association of dietary fibre with reduced mortality from cancer. As with other human-based studies on dietary fibre, there remains a question regarding causality, and other healthy lifestyle factors that associate with increased intake of dietary fibre may mediate at least some of the apparent benefits of dietary fibre on CVD risk. Neither, however, is it possible to disprove an important role for dietary fibre as a direct causal factor for improved CVD outlook. Indeed, the association of reduced levels of plasma Trimethylamine-N-oxide (TMAO, a by-product of certain gut microbiota, derived from choline) with reduced risk for CVD provides one potential mechanistic explanation for the association of dietary fibre intake with reduced CVD risk [78]. Although further RCTs on the direct benefits of dietary fibre (and the elucidation of the underlying mechanisms that mediate such benefits) would be desirable, based on currently reported data, the widely accepted dietary advice to optimise dietary fibre intake within the population seems entirely justified, realistic and safe.

### 5.8. Colorectal Carcinoma (CRC) Prevention

Colorectal carcinoma (CRC) is the third most common cancer globally [79]. Gianfredi and colleagues published a systematic review and meta-analysis to explore the association between dietary fibre and risk of CRC through a comparison between individuals with the highest and lowest dietary fibre intake. Of 376 papers, 25 datasets were included in the analysis, which revealed a protective role of dietary fibre intake on risk of CRC, with a highly significant effect size of 0.74 (95% CI: 0.67–0.82) and a moderate level of statistical heterogeneity [79]. A further case-control study on the effects of dietary fibre on CRC in a Chinese population of >260 cases and >250 controls was reported by Song and colleagues [80]. In this study, compared with CRC cases, the control group consumed significantly more vegetables, soy food and total fibre. There were significant inverse associations between risk for CRC and total fibre intake and consumption of vegetable fibre, soluble and insoluble fibre. Interestingly, consumption of fruit, meat and sea-food were equivalent between cases and controls [80]. In a further review of the existing literature, with identification of RCTs on the effect of dietary fibre on the recurrence of colorectal adenomatous polyps in those with a history of adenomatous polyps, and incidence of CRC compared with placebo, Yao and colleagues used a fixed-effect model based on >4700 participants from seven studies [81]. The authors showed no evidence to support dietary fibre intake in the prevention of recurrence of adenomatous polyps in those with a history of adenomatous polyps over a 2–8-year duration, although an attrition bias may have affected the data [81]. Based on current evidence, a protective role for dietary fibre on the development of CRC seems likely, although there is a need for more focused and prospectively designed RCTs to provide further evidence to support advocating a high fibre diet for prevention of CRC in the general population, particularly those with existing colonic adenomatous disease.

### 5.9. Mortality

In one meta-analysis based on seven prospective cohort studies, there was an 11% reduction (95% CI 0.85–0.92) in all-cause mortality for each 10-g per day increment in the consumption of dietary fibre [82]. Regarding the type of dietary fibre, it appeared that cereal fibre consumption associated with the strongest inverse association with mortality [82]. It should be noted, however, that mortality as an outcome is not ideal as a marker of disease prevention [19]. Furthermore, the data presented here are based on association and do not provide evidence that increased dietary fibre necessarily causes an improved mortality rate. It remains possible that factors related to increased fibre intake, including healthy lifestyle factors (such as a healthy diet generally, physical activity and sleep sufficiency), may mediate at least some of the association between increased dietary fibre intake and improved longevity.

Having explored the multiple and varied mechanisms whereby dietary fibre may confer its numerous health benefits (summarised in Table 1), including the important associations between dietary fibre intake and body weight and overall metabolic health, it is important to address the apparent disconnection between such benefits and the impoverishment of fibre consumption that typifies our modern-day Westernised diets. It is also important to explore how we can optimise fibre consumption at a population level in the future.

## 6. Optimisation of Dietary Fibre Intake for the Future

Our modern-day lifestyles within Westernised countries differ radically from our hominid hunter-gatherer ancestors. Accordingly, our genetic make-up is maladapted to our modern-day environs and lifestyles. Furthermore, our gut microbiota, that co-evolved with us over millions of years, and upon which our health utterly relies [55], have likely changed radically through our evolutionarily highly unusual modern-day lifestyles, a major component of which is our diet.

As outlined earlier, our modern-day diets within European countries and North America are impoverished of fibre [19]. One obvious factor relates to the highly processed diets that many people adopt. Underlying explanations for such a radical shift in dietary consumption in recent decades are likely multi-factorial. These include an abundance of cheap and highly processed food supplies within our supermarkets and a bias for such food production from many food companies. Further contributing factors include advertising for processed foods, cultural and societal changes, convenience of highly processed diets (with a reduced need for cooking from raw ingredients), highly abundant ‘fast-food’ outlets and the hedonic and potentially addictive effects of the unnatural sugar–fat combination that typifies many highly processed foods [83]. One problem with highly processed foods is that their fibre content tends to be lower than meals prepared from raw ingredients. Rauber and colleagues reported on the consumption of ultra-processed foods within the UK, based on cross-sectional data from the UK National Diet and Nutrition Survey between 2008 and 2014 [84]. Based on a four-day food diary, it was shown that just 30.1% of calories originated from unprocessed or minimally processed foods, with the majority of calories (56.8%) coming from ultra-processed foods. Furthermore, whilst consumption of ultra-processed foods was associated with increased intake of carbohydrates, free sugars, saturated fats and sodium, consumption of ultra-processed foods also inversely correlated with dietary fibre intake [84].

To address the lack of dietary fibre within Westernised populations will require a multi-faceted approach from multiple angles. Lifestyle and dietary advice and education on its own is simply not effective, as evidenced by the fact that despite decades of healthy eating advice, including encouragement to eat more fibre (with much media interest in fibre intake, such as brown versus white bread), our diet still lacks sufficient fibre content. In our view, for population-based behaviour change to occur, we need to address key factors, as outlined here.

### 6.1. Availability of High-Fibre Food

Many of us buy our food from supermarkets [85]. The convenience of shopping in a supermarket and the time- and cost-saving compared with shopping in more traditional retail outlets aligns well with our busy, time-pressured modern daily lives. Therefore, what is available to us when we shop in supermarkets heavily influences what we eat. Apart from the fruit and vegetable isles that usually confront us on entering a supermarket, much of the rest of the food on offer is processed in some way. To improve our fibre consumption would require increased availability of non-processed foods in our supermarkets, with a corresponding decrease in the availability of highly processed foods. This simple strategy is likely to encourage many of us to eat more fibre. Furthermore, a wider choice of high-fibre foods should be available (brown breads and pastas for example), with clear labelling on packaging to indicate these healthier higher-fibre options.

### 6.2. Manufacture of High-Fibre Food

Supermarkets can only sell a wider variety of high-fibre foods if their suppliers, primarily the food companies, produce more high-fibre food options. Whilst it would perhaps be naïve to imagine a scenario where processing of foods was eliminated entirely (given the need for at least some processing to improve food longevity, for example), it would be desirable for food companies to continue to reduce the sugar–fat content of processed foods, for example. Whilst much progress has been made in this regard in recent years (including the availability of low-sugar ‘diet’ drinks [86] and foods), there is still much progress that should be made. Regarding population-wide optimisation of dietary fibre intake, the main strategy over recent decades appears to have been through provision of dietary education and public-health messages, with an attempt to change mind-sets. Unfortunately, this strategy does not seem to have worked. There is corroboration of this conclusion by reported data from studies on dietary fibre. For example, in our own interventional OptiFiT study, the control group was encouraged to eat more fibre with little success [34,35]. An alternate strategy would be for the food industries to produce healthy processed foods that are rich in dietary fibre. There is no reason why processed foods should not contain increased amounts of fibre—for example, through addition of powdered chicory, broccoli or whole grains to certain foods [21]. In a future scenario, with addition of small amounts of fibre to many types of processed foods, and for this process to become ubiquitous and normalised, dietary fibre intake within the population would increase without the need to change the mind-set of the consumer. The population-wide health benefits would likely be substantial (particularly for less educated people who are generally at increased risk of health problems), based on the wealth of evidence outlined in this review.

### 6.3. Cultural and Societal Factors

Perhaps most intransigent to change is our culture and society. Any cultural change is usually a long process, perhaps even over a generation, and is unlikely to happen overnight. In addition to an effective education program on the health benefits of a high fibre diet, we need to develop imaginative strategies to encourage us to eat more fibre. Perhaps development of new recipes that use cauliflower rice instead of white rice or recipes with the addition of powdered broccoli, for example. Educating our children is important [87] and we need to incorporate digital means to facilitate such changes. Perhaps an app that counts our daily intake of fibre and provides us with rewards on achieving our goals, or for a ‘fibre counter’ incorporated into a fitness tracker (to be used alongside data on step count, which many of us seem to find motivating). With government backing and a unified approach from supermarkets and food companies, cultural and societal change would likely follow on naturally and would be much more difficult to effect without these other necessary changes.

There are limiting factors that conspire against the establishment of optimised dietary fibre intake for the populace. These include the taste, appearance and digestibility of certain fibre-rich foods (primarily fermentable fibre), which some people may not prefer. Furthermore, some people avoid fibre-rich food because of its association with intestinal gas production and its consequences. These factors may explain at least some of the overall resentment towards fibre-rich foods within the general population. Furthermore, there may be important advantages for intake of non-soluble and less fermentable fibres (as we used in our reported studies [36]), including a relatively low cost and the versatility for addition of such non-soluble fibre in small quantities to many foods. Indeed, in the UK, the non-soluble fibres that we used in our studies [36] are currently available as a toast bread with added fibres. Furthermore, small amounts of non-soluble fibres (up to around 3%) are almost unremarkable in many products and would optimise fibre intake without adding any calories. This is important, as reliance for the recommended and desired daily dietary fibre intake from cereal products alone (which typically contain around 10% fibre) would require the consumption of around 300 g per day, which would exceed daily caloric requirements.

Other limitations of reported studies on dietary fibre include potential ceiling effects for the health benefits of dietary fibre, including for reducing the risk of cardiovascular disease and T2D. Many dietary fibre-related studies also have limitations in the quality of the data reported, with most studies being observational with relatively few participants and/or for a short duration. These factors, along with methodological heterogeneity between reported studies, and a lack of suitable outcomes provide a rationale for the implementation of well-powered and well-designed, blinded (through usage of supplements or fortification as placebo arms), RCTs on the effects of dietary fibre on clinically-relevant health outcomes.

## 7. Concluding Remarks

To conclude, much evidence supports an important role for dietary fibre intake as a contributor to overall metabolic health, through key pathways that include insulin sensitivity. Furthermore, there are clear associations between dietary fibre intake and multiple pathologies that include cardiovascular disease, colonic health, gut motility and risk for CRC. Dietary fibre intake also correlates with mortality. The gut microflora functions as an important mediator of the beneficial effects of dietary fibre, including the regulation of appetite and metabolic processes and chronic inflammatory pathways.

Many factors contribute towards the impoverishment of dietary fibre intake in the typical Western diet. Unfortunately, there is habituation of many of us to our modern-day environs, lifestyles, diets and eating-related behaviours. The problem is that what most of us consider normal is actually highly abnormal and about as far away from what our hominid hunter-gatherer ancestors experienced and enjoyed as it is possible to imagine. The fact is that over decades, a blink of an eye in hominid history, we have gradually migrated into our current environments, lifestyles and culture, and it is very hard for most of us to imagine an alternate scenario. Stepwise changes are required. By adopting some of the suggested strategies here, it is our belief that real change in dietary fibre intake can occur. Despite all the technological breakthroughs and progress of our species during much of the 20th century and the resultant radical changes in our lifestyles, environments and eating habits, we may spend much of the 21st century searching for ways in which to re-establish some healthier lifestyle choices. Some of these will, doubtless, include lifestyle choices that our ancestors enjoyed in the pre-industrialised era, including, fundamentally, the adoption of a non-processed diet that is full of fibre.

Consumption of ultra-processed foods is not natural. We did not evolve to adopt this dietary maladaptive behaviour and neither did our gut flora. Our hominid ancestors and all of their ancient ancestors never ate ultra-processed foods. Our current dietary habits in the Western world are extremely unusual and will even be considered as an anomaly by food historians from a future era. Whilst global poverty and ill health remains a major problem and concern, most Western societies have developed shelter, clean water, sanitation and advanced healthcare systems to improve our comfort, quality of life and overall longevity. Our modern Western diets, however, remain a problem. Unlike the other examples of human advanced development provided here, the clear benefits of which are incontrovertible, our diets seem to have deteriorated. This is unfortunate given the central role of our diet, including the multiple benefits mediated by our gut microbiota, as an important determinant of overall health and wellbeing. Although many aspects of our diet are concerning (including our excessive consumption of sugars and fat), the clear lack of dietary fibre in our modern-day diet is of particular concern.

Despite a wealth of evidence generated over many decades to corroborate the multiple health benefits of dietary fibre, the health risks of a diet that lacks fibre and the corresponding efforts to provide public health messages to educate the populace, sadly, within the Western world, our diets remain lacking in fibre. It would be easy to apportion blame solely on the food companies that process fibre-impoverished food products. This would be wrong: we all have a choice regarding our diet, although it is unfortunate that a healthy diet generally costs around 25–30% more than an unhealthy diet based on highly processed foods. However, the wide availability, convenience and even relatively low cost of highly processed foods in our supermarkets should not compel us to make those relatively unhealthy choices. As food consumers, our choice of high-fibre foods in preference to fibre-impoverished ultra-processed foods likely has a major positive impact on our future health and wellbeing and will ultimately influence the strategic commercial plans of food companies, with likely future improvements in the fibre content of processed food production. In our capitalist culture of modern Westernised societies with ‘consumer as king’, we all need to vote with our mouths, and in the process, re-discover the joy of cooking with fresh and fibre-replete ingredients.

## Figures and Tables

**Table 1:** Summary of the health benefits of dietary fibre.

Effect	Health Benefit
Metabolic	Improved insulin sensitivity (mainly insoluble fibres), reduced risk of developing T2D (mainly insoluble cereal fibres and whole grains) Improved glycaemic status and lipid profiles (mainly soluble fibres), reduced body weight and abdominal adiposity
Gut microflora	Gut microbial viability and diversity, metabolites from gut microflora (including SCFAs)
Cardiovascular	Chronic inflammation, cardiovascular risk, mortality
Depression	Chronic inflammation, gut microbiota
Gastrointestinal Localised	Colonic health and integrity, colonic motility, colorectal carcinoma

SCFAs = Short Chain Fatty Acids; T2D = Type 2 Diabetes Mellitus.
